# The Involvement of NFAT Transcriptional Activity Suppression in SIRT1-Mediated Inhibition of COX-2 Expression Induced by PMA/Ionomycin

**DOI:** 10.1371/journal.pone.0097999

**Published:** 2014-05-23

**Authors:** Yu-Yan Jia, Jie Lu, Yue Huang, Guang Liu, Peng Gao, Yan-Zhen Wan, Ran Zhang, Zhu-Qin Zhang, Rui-Feng Yang, Xiaoqiang Tang, Jing Xu, Xu Wang, Hou-Zao Chen, De-Pei Liu

**Affiliations:** 1 State Key Laboratory of Medical Molecular Biology, Department of Biochemistry and Molecular Biology, Institute of Basic Medical Sciences, Chinese Academy of Medical Sciences & Peking Union Medical College, Beijing, People's Republic of China; 2 State Key Laboratory of Medical Molecular Biology, Department of Medical Genetics, Institute of Basic Medical Sciences, Chinese Academy of Medical Sciences & Peking Union Medical College, Beijing, People's Republic of China; Univ. Kentucky, United States of America

## Abstract

SIRT1, a class III histone deacetylase, acts as a negative regulator for many transcription factors, and plays protective roles in inflammation and atherosclerosis. Transcription factor nuclear factor of activated T cells (NFAT) has been previously shown to play pro-inflammatory roles in endothelial cells. Inhibition of NFAT signaling may be an attractive target to regulate inflammation in atherosclerosis. However, whether NFAT transcriptional activity is suppressed by SIRT1 remains unknown. In this study, we found that SIRT1 suppressed NFAT-mediated transcriptional activity. SIRT1 interacted with NFAT, and the NHR and RHR domains of NFAT mediated the interaction with SIRT1. Moreover, we found that SIRT1 primarily deacetylated NFATc3. Adenoviral over-expression of SIRT1 suppressed PMA and calcium ionophore Ionomycin (PMA/Io)-induced COX-2 expression in human umbilical vein endothelial cells (HUVECs), while SIRT1 RNAi reversed the effects in HUVECs. Moreover, inhibition of COX-2 expression by SIRT1 in PMA/Io-treated HUVECs was largely abrogated by inhibiting NFAT activation. Furthermore, SIRT1 inhibited NFAT-induced COX-2 promoter activity, and reduced NFAT binding to the COX-2 promoter in PMA/Io-treated HUVECs. These results suggest that suppression of NFAT transcriptional activity is involved in SIRT1-mediated inhibition of COX-2 expression induced by PMA/Io, and that the negative regulatory mechanisms of NFAT by SIRT1 may contribute to its anti-inflammatory effects in atherosclerosis.

## Introduction

The nuclear factor of activated T cells (NFAT) family of transcription factors includes NFATc1, NFATc2, NFATc3, NFATc4 and NFAT5. NFATc1 to NFATc4 are regulated by calcium-calcineurin (CnA) signaling. Previous studies have observed phosphorylation and nuclear translocation of NFAT in endothelial cells exposed to agents stimulating CnA-NFAT signaling such as phorbol 12-myristate 13-acetate (PMA) and calcium ionophoreionomycin (Io) [1], [2]. CyclosporinA (CsA) is a potentinhibitor of CnA-NFAT signaling. Based on the inhibitory effects of CsA on gene expression, several potential CnA-NFAT target genes were described in activated endothelial cells, including cyclooxygenase (COX)-2 [3], granulocyte-macrophage-colony-stimulating factor (GM-CSF) [1], interleukin 8 (IL-8) [4], and tissue factor (TF) [5]. Prostaglandins formed by inducible COX-2, including PGI_2_ in endothelial cells, mediate the development of classical signs of inflammation such as leukocyte activation, vasodilatation, pain and oedema [6], [7]. Endothelium-targeted actions of selected agents that reduce inflammation, such as non-steroidal anti-inflammatory agents (ibuprofen, ASA, celecoxib), targeted COX-2 have been used to alleviate inflammation of clinical indication [8]. Although NFAT is essential in the process of inflammation, the mechanism underlying negative regulation of NFAT in endothelial cells remains unknown.

Sirtuin 1 (SIRT1), a mammalian homolog of yeast Sir2, is an NAD^+^-dependent class III histone deacetylase. SIRT1 plays critical roles in aging, cell cycle regulation, apoptosis and inflammation [9], [10]. SIRT1 is expressed highly in the vasculature and acts as a key regulator of vascular endothelial homeostasis by controlling angiogenesis, vascular tone and endothelial dysfunction [11], [12]. Our previous studies have demonstrated that SIRT1over-expression in endothelial cells decreases atherosclerosis and protects against hyperglycemia-induced endothelial dysfunction [13], [14]. SIRT1 interacts with a number of non-histone substrates including the forkhead box o (Foxo), p53 and Activator protein-1 (AP-1) transcription factors, which mediate specific downstream functions of SIRT1 [15]–[18].

Although SIRT1 prevents endothelial superoxide production, inhibits NF-κB signaling, and diminishes expression of adhesion molecules [19], little is known about whether SIRT1 regulates endothelial inflammation via transcriptional suppression of NFAT. In the present study, we found that SIRT1 suppressed NFAT transcriptional activity and interacted with NFAT. Moreover, inhibition of COX-2 expression by SIRT1 in PMA/Io treated HUVECs was largely abrogated by inhibiting NFAT activation. Furthermore, SIRT1 inhibited NFAT induced COX-2 promoter activity, and reduced NFAT binding to the COX-2 promoter in PMA/Io treated HUVECs. Taken together, these findings indicate that the negative regulatory mechanisms of NFAT by SIRT1 may contribute to its anti-inflammatory effects in atherosclerosis.

## Materials and Methods

### Plasmids

Human NFATc1, NFATc2 and NFATc3 full-length cDNA were obtained by reverse transcriptase-PCR (RT-PCR) from Jurkat cells. The HA-tagged NFATc1, NFATc2 and NFATc3 expression vectors were constructed by inserting human NFATc1, NFATc2 and NFATc3 full-length cDNA into the pCMVHA vector. The primer pairs were listed in Table S1. The Myc-tagged NFATc1, NFATc2 and NFATc3 expression vectors were constructed by inserting human NFATc1, NFATc2 and NFATc3 full-length cDNA into the pcDNA4b vector. The primer pairs were listed in Table S2. The HA-tagged NFATc3 (1-407AA), (408-674AA), (675-1075AA), (1-674AA) and (408-1075AA) expression vectors were constructed by inserting the truncated NFATc3 fragments into the pCMVHA vector.The primer pairs were listed in Table S3.

The NFAT-luciferase report plasmid was obtained by the insertion of a double-stranded oligonucleotide representing three NFAT DNA binding sites into the pTA-luciferase plasmid. SIRT1 expression vectors were gifts from Dr. Ishikawa [20]. The mouse COX-2 promoter plasmid containing a 1068-bp fragment, −1003 to +65 relative to the transcription start site, was subcloned into pGL3 vector (COX-2-Luc) [18]. The full-length HA-c-Jun or HA-c-Fos expression vectors were constructed by inserting the human c-Jun or c-Fos cDNA into the pcDNA3.1 vector [18].

### HUVEC Culture and Adenovirus Generation

Endothelial cells were freshly isolated from human umbilical cord veins as previously described [13] and cultured in M200 medium. Cells between the third and the sixth passages were grown in monolayers in a humidified atmosphere of 5% CO_2_ at 37°C, and used for experiments at >80% confluence. Replication-defective adenoviral vectors expressing SIRT1 (Ad-SIRT1) or control green fluorescentprotein (Ad-GFP) were prepared using the AdEasy vector kit (Quantum Biotechnologies) in according to the manufacturer's instructions. The adenovirus-mediated knockdown of SIRT1 (Ad-SIRT1 RNAi) and control vector (Ad-U6) were generated using the same system. The SIRT1 RNAi sequence was reported previously [21]. HUVECs were infected with the above adenovirus for 24 h and were cultured in fresh M200 for further treatment. PMA, Ionomycin, cyclosporin A, resveratrol, sirtinol, NFAT inhibitor, and NF-κB inhibitor JSH-23 were purchased from Sigma-Aldrich.

### Real-Time PCR

Total RNA was extracted from cells using TRIzol reagent (Invitrogen) in according to the manufacturer's instructions. Two micrograms of total RNA wereused to synthesize first-strand cDNA with MuLV reverse transcriptase (New England BioLabs) using random primers. The genes were examind by real-time PCR using the BioRad iCycler iQ5 Real-Time PCR Detection System with the SYBR Premix EX Taq™ (TaKaRa). The fluorescence curves were analyzed with iCycler iQ5 Optical System Software (Version 2.0). β-Actin mRNA was used as internal control for normalizing gene expression. The real-time PCR primer pairs were listed in Table S4.

### Western Blotting

Cellular protein was extracted with RIPA buffer (25 mM Tris-HCl pH 7.6, 150 mM NaCl, 1% NP-40, 1% sodium deoxycholate, 0.1% SDS). After complete homogenization on ice, samples were sonicated and centrifuged at 4°C. The supernatants were transferred into fresh tubes and protein concentrations were detected by the BCA method. Equal amounts of protein (20 µg/lane) were separated by SDS-PAGE and transferred onto polyvinylidenedifluoride membranes (Millipore). After blocking with 5% non-fat milk, the filters were incubated overnight at 4°C with the primary antibodies against HA (Sigma-Aldrich), β-actin (Sigma-Aldrich), GAPDH (Cell Signaling Technology), Myc (Santa Cruz), human SIRT1 (Santa Cruz), Anti-acetyl-lysine (Millipore) and COX-2 (Cayman Chemicals). After washing and incubating with the appropriate horseradish-peroxidase-conjugated secondary antibody (Santa Cruz), the immune complexes were visualized with a chemiluminescent reagent. Western blots were quantified by the intensity values and were normalized to GAPDH or β-actin.

### Co-Immunoprecipitation

The cells were collected, and the proteins were solubilized in IP buffer (50 mM Tris pH 8.0, 150 mM NaCl, 1% protease inhibitor mixture). One milligram of lysed protein was incubated with specific antibodies and precipitated with protein A- or G-agarose (Upstate Biotechnology). Precipitated proteins or total lysate swere separated in 12% PAGE followed by western blotting with specific antibodies. The blots were then incubated with a horseradish-peroxidase-conjugated secondary antibody and exposed to ECL reagent for detection of protein expression.

### Immunofluorescence

HUVECs grown on 1% gelatin-coated coverslips in 24-well tissue culture plates were either untreated or incubated with PMA (10 ng/ml) and Io (0.25 µM) for 1 h. The cells were fixed with 4% paraformaldehyde in PBS for 15 min at room temperature and washed three times (5 min each) with PBS, then incubated with 0.5% Trition X-100 in PBS for 10 min at room temperature. After blocking with 3% BSA in PBS for 60 min at room temperature, the coverslips were incubated with anti-NFATc3 (Santa Cruz) and anti-SIRT1 (Millipore) antibodies overnight at 4°C. Unbound antibody was removed by rinsing three times (5 min each) with PBS at room temperature, and the coverslips were incubated for 1 h at room temperature with Alexa Fluor 488 Donkey Anti-Rabbit IgG (H+L) Antibody (Invitrogen) or Alexa Fluor 594 Donkey Anti-Mouse IgG (H+L) Antibody (Invitrogen). After washing three times (5 min each) with PBS, and mounted in DAPI (Invitrogen) on glass slides. The cells were visualized with a confocal fluorescence microscope.

### Transfection and Luciferase assay

HEK293 cells were transfected with Lipofectamine 2000 (Invitrogen) in according to the manufacturer's instructions. Luciferase assays were performed using a dual luciferase reporter assay system (Promega). Luciferase activity was normalized by transfection efficiency using pRL-TK reporter as an internal control. The results are expressed as percentages of relative luciferase activity of the control group.

### Chromatin Immunoprecipitation (ChIP)

HUVECs infected with adenoviral vectors encoding SIRT1 (Ad-SIRT1) or control Ad-GFP followed by incubation of PMA (10 ng/ml) and Io (0.25 µM) for 3 h were treated with 1% formaldehyde for 15 min. The cross-linked chromatin was sheared by sonication, and the sonicated complex was used for immunoprecipitation with specific antibodies or nonimmune rabbit IgG (Santa Cruz) as control. Immunoprecipitated complexes were collected by using Dynabeads Protein A (Invitrogen). The immunoprecipitated chromatin DNA was amplified by real-time PCR using primers for COX-2 promoter region or 3′-UTR. The real-time PCR primer pairs were listed in Table S5. Antibodies for NFATc3 (Santa Cruz) and NF-κB p65 (Millipore) were used in ChIP assay.

### Statistical Analysis

All results are expressed as means ± SD of three independent experiments unless otherwise stated. Statistical analyses were performed by two-tailed unpaired Student's t-tests to determine statistical significance between the groups. P≤0.05 was considered significant.

## Results

### SIRT1 suppresses NFAT transcriptional activity

**Figure 1:**
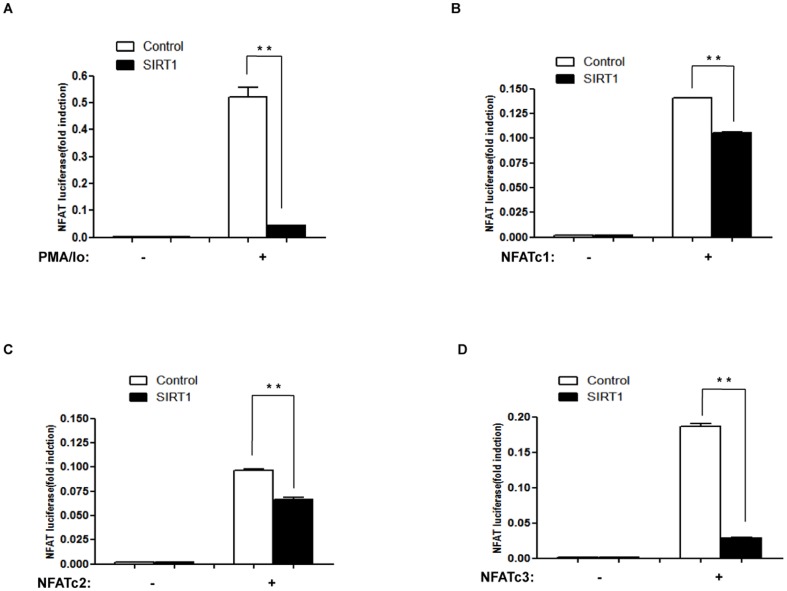
SIRT1 suppresses NFAT transcriptional activity. (A) HEK293 cells were transfected with 0.1 µg NFAT luciferase reporter (NFAT-luc), 30 ng pRL-TK, and 0.3 µg SIRT1 expression vector or control (pcDNA3.1) for 24 h and treated with PMA (10 ng/ml) and Io (0.25 µM) or vehicle for another 3 h. Luciferase activities are presented as the means ± standard deviation (S.D.) of triplicate samples and are representative of three independent experiments. **, p≤0.01 versus control. (B–D) HEK293 cells were transfected with 0.1 µg NFAT luciferase reporter (NFAT-luc), 30 ng pRL-TK, 0.3 µg SIRT1 expression vectors or a control (pcDNA3.1), and 0.3 µg NFATc1 (B), or NFATc2 (C), or NFATc3 (D) expression vectors for 24 h. Luciferase activities are presented as the means ± standard deviation (S.D.) of triplicate samples and are representative of three independent experiments. **, p≤0.01 versus control.

To determine whether SIRT1 suppresses the transcriptional activity of NFAT, we performed luciferase assays using NFAT-luciferase reporter. HEK 293 cells were transfected with NFAT- luciferase reporter (NFAT-luc), pRL-TK reporter (internal control), either SIRT1 or control vector pcDNA3.1 and then were treated with PMA and ionomycin (PMA/Io). PMA/Io induced NFAT transcriptional activity, which was inhibited by over-expression of SIRT1 (Figure 1A).

This study focuses on the three calcium-regulated NFAT proteins that are expressed in endothelial cells (Figure S1): NFATc1, NFATc2 and NFATc3. We further transfected HEK293 cells with NFAT luciferase reporter (NFAT-luc), pRL-TK reporter (internal control), SIRT1 and NFAT expression vectors. We found that NFAT transcriptional activity was suppressed by over-expression of SIRT1 (Figure 1B, 1C, 1D).

We also tested whether the other sirtuin family member SIRT2 suppresses NFAT transcriptional activity, we transfected HEK293 cells with NFAT luciferase reporter (NFAT-luc), pRL-TK reporter (internal control), SIRT2 and NFAT expression vectors. In contrast to SIRT1, SIRT2 had no obvious influence on transcriptional activity of NFAT (Figure S2).

### SIRT1 interacts with NFAT

**Figure 2:**
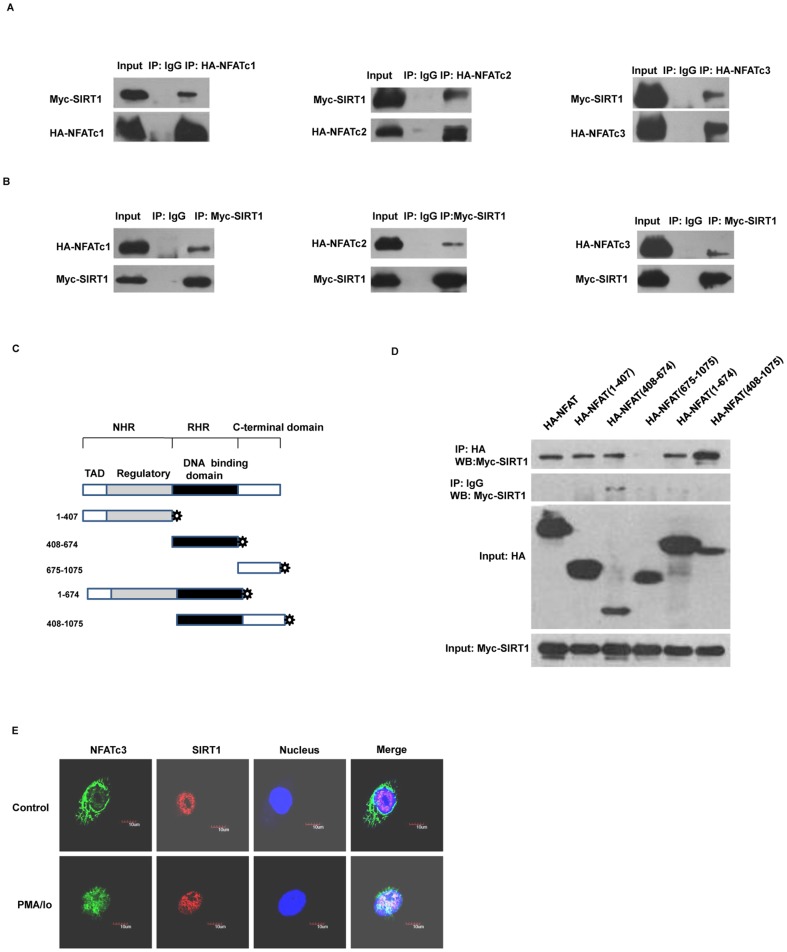
SIRT1 interacts with NFAT. (A, B) HEK 293 cells were co-transfected with a Myc-tagged of SIRT1 and HA-tagged of NFAT expression vectors, and were analyzed using co-immunoprecipitation and western blotting. The images are representative of three independent experiments. (C) Schematic diagram of full-length or truncated NFATc3 is shown. NHR, NFAT homology region; RHR, Rel homology region; TAD, transactivation domain. (D) HEK293 cells were transfected with HA-tagged either full-length or truncated NFATc3 and Myc-tagged SIRT1 expression vectors for 24 h and were lysed for co-immunoprecipitation and western blotting. The images are representative of three independent experiments. (E) HUVECs were treated with PMA (10 ng/ml) and Io (0.25 µM) or vehicle DMSO for 1 h. Immunofluorescence experiments were done with anti-NFATc3 and anti-SIRT1. The nuclei were stained with DAPI. The cells were analyzed by confocal fluorescent microscopy. The scale bar represents 10 µm. The images are representative of three independent experiments.

Because SIRT1 usually exerts biological functions mediated by transcription factors through interacting and deacetylating, we investigated whether SIRT1 interacted with and deacetylated NFAT. We performed co-immunoprecipitation (Co-IP) experiments in HEK293 cells that expressed hemagglutinin epitope (HA) -tagged NFAT and Myc-tagged SIRT1. We found obvious interaction between SIRT1 and NFAT (Figure 2A and 2B).

The conserved core region of NFAT proteins consists of two tandem domains: a regulatory domain, which is also known as the NFAT-homology region (NHR); and the Rel homology region (RHR), which binds DNA [22]. To further determine the domain of NFAT that is required for the interaction with SIRT1, we generated expression vectors containing HA-tagged full-length or truncated NFATc3 mutants (NHR, RHR, and C-terminal domain) (Figure 2C). HA-tagged full length or truncated NFATc3 were co-transfected with Myc-tagged SIRT1 to HEK293 cells. The Co-immunoprecipitation results showed that the NHR and RHR domains of NFATc3 were responsible for the interaction with SIRT1 (Figure 2D).

Next, we tested whether endogenous SIRT1 could co-localize with NFATc3 in vivo. Immunofluorescence with anti-NFATc3 in HUVECs revealed that, in the absence of PMA/Io, NFATc3 was localized in the cytoplasm. However, PMA/Io triggered the translocation of NFATc3 from the cytoplasm to the nucleus. In contrast to NFATc3, SIRT1 always located in nucleus, either in the absence or presence of PMA/Io. After treatment of PMA/Io, NFATc3 and SIRT1 co-localized within the nucleus (Figure 2E). These results suggest that endogenous SIRT1 interacts with NFATc3 in PMA/Io-treated HUVECs.

### NFATc3 is primarily deacetylated by SIRT1

**Figure 3:**
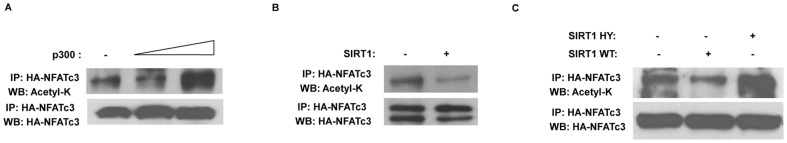
NFATc3 is primarily deacetylated by SIRT1. HEK293 cells were transfected with: (A) HA-tagged NFATc3 and either p300 or control (pcDNA3.1) vectors for 24 h; (B) HA-tagged NFATc3 and either SIRT1 or control (pcDNA3.1) vectors for 24 h; (C) HA-tagged NFATc3 and wild-type SIRT1 (SIRT1 WT) or dominant negative type (SIRT1H363Y) or control (pcDNA3.1) vectors for 24 h. HA-tagged NFATc3 was then immunoprecipitated and the acetylation of NFATc3 was assessed by western blotting using anti-acetylated lysine antibody. Total levels of NFATc3 were assessed with anti-HA antibody. The images are representative of three independent experiments.

NFATc2 is trans-activated and acetylated by p300 [23], [24]. Thus, we detected whether NFATc3 is also acetylated by p300. HEK 293 cells were co-transfected with HA-tagged NFATc3 and either p300 or control vector pcDNA3.1. Similar with NFATc2, NFATc3 was also acetylated by p300 (Figure 3A). To further determine whether NFATc3 is deacetylated by SIRT1, HEK 293 cells were co-transfected with HA-tagged NFATc3 and either SIRT1 or control vector pcDNA3.1. The result showed that NFATc3 was primarily deacetylated by SIRT1 (Figure 3B). In contrast to NFATc3, SIRT1 had no obvious influence on acetylation level of NFATc1 and NFATc2 (Figure S3).

Moreover, to determine whether the acetylation level of NFATc3 is dependent on SIRT1 deacetylase activity, HEK 293 cells were co-transfected with HA-tagged NFATc3 and wild type SIRT1 (SIRT1 WT), or dominant negative SIRT1 (SIRT1H363Y), or control vector pcDNA3.1, respectively. We found that NFATc3 was not deacetylated by SIRT1H363Y (Figure 3C). The immunoprecipitation results suggest that SIRT1-mediated deacetylation of NFATc3 was dependent on its deacetylase activity.

### SIRT1 does not interfere with the interaction between NFAT and c-Jun/c-Fos

**Figure 4:**
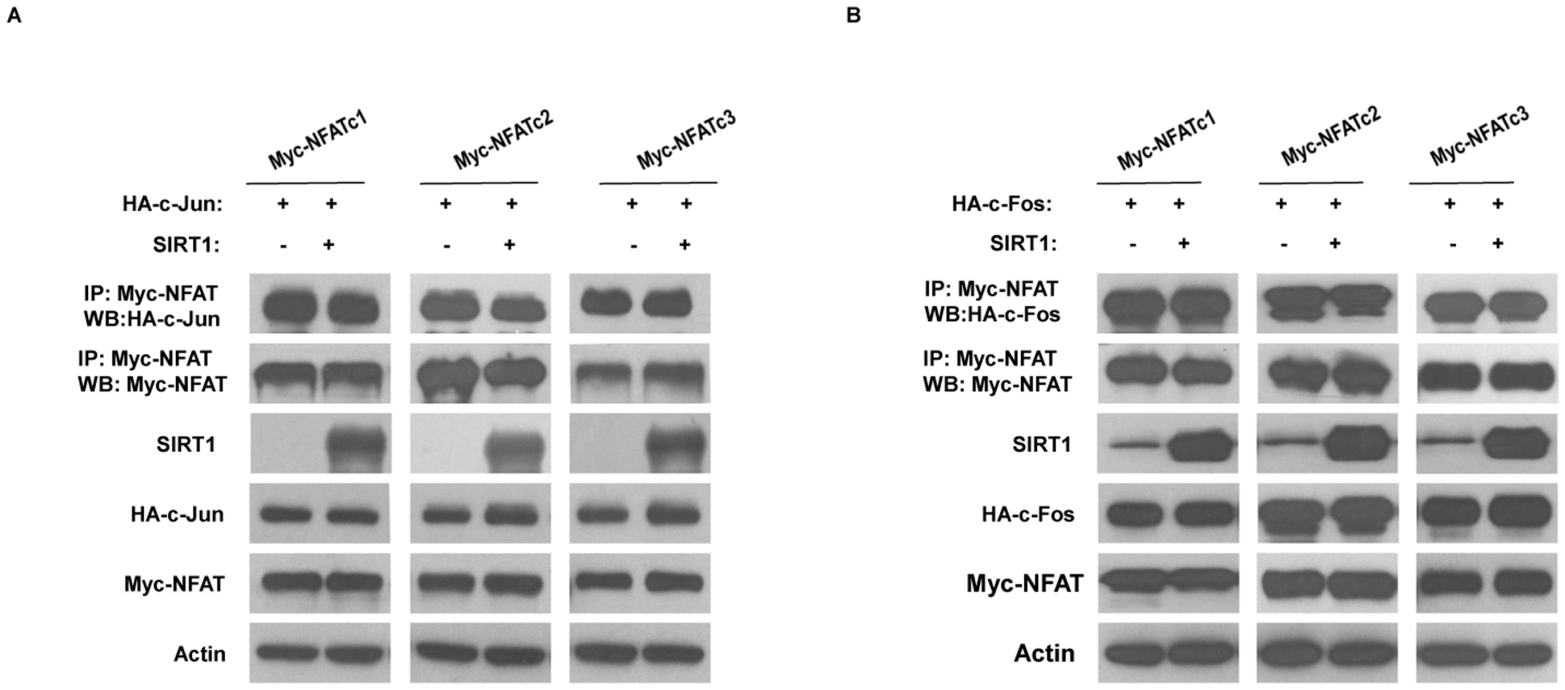
SIRT1 does not interfere the interaction between NFAT and c-Jun or c-Fos. (A) HEK293 cells were transfected with the HA-tagged c-Jun, Myc-tagged NFAT, either SIRT1 or control (pcDNA3.1) vectors for 24 h. Myc-tagged NFAT was then immunoprecipitated and c-Jun was assessed by western blotting using anti-HA antibody. The images are representative of three independent experiments. (B) Similar studies were performed using HA-tagged c-Fos. The images are representative of three independent experiments.

Our previous work demonstrated that SIRT1 interacted with and deacetylated c-Jun and c-Fos (Activator protein-1, AP-1) [18]. AP-1 proteins are the main transcriptional partners of NFAT [25]. To determine whether SIRT1 interferes the interaction between NFAT and AP-1, we performed co-immunoprecipitation experiments in HEK293 cells that expressed either HA-tagged c-Jun or c-Fos, Myc-tagged NFAT, and SIRT1 expression vectors. The Co-immunoprecipitation results showed that SIRT1 did not interfere the interaction between NFAT and either c-Jun or c-Fos (Figure 4A, 4B).

### NFAT is involved in the inhibition of COX-2 expression by SIRT1 in PMA/Io-treated HUVECs

**Figure 5:**
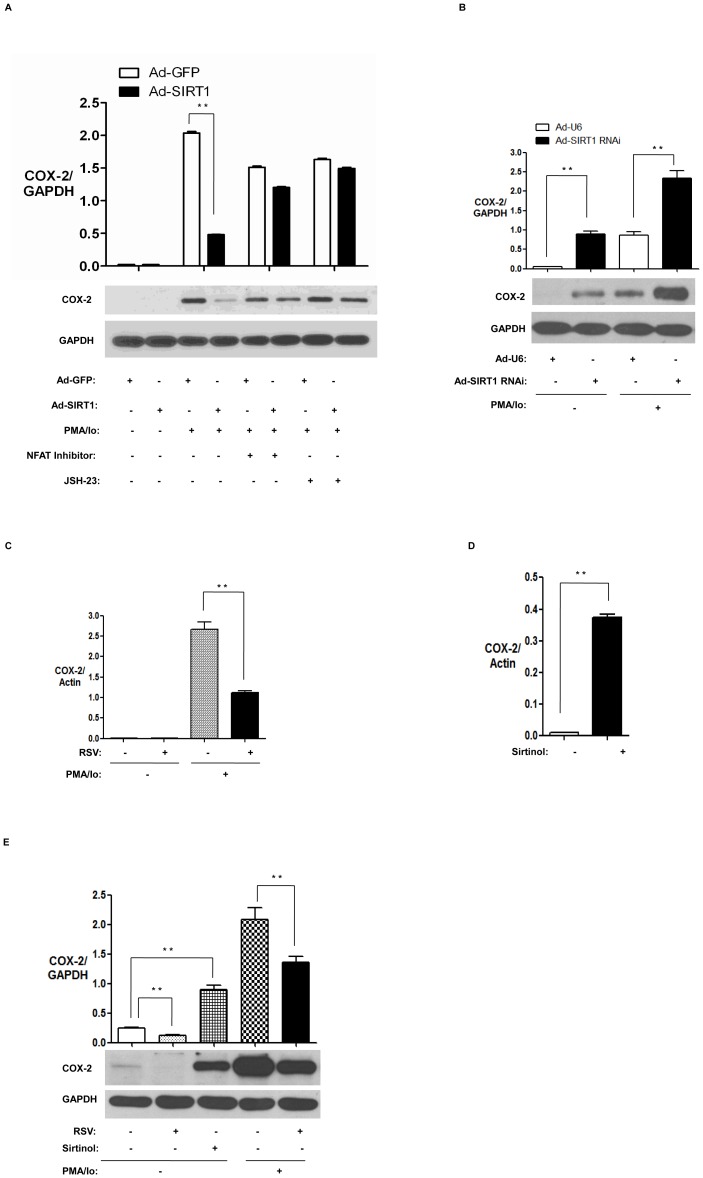
NFAT is involved in the inhibition of COX-2 expression by SIRT1 in PMA/Io-treated HUVECs. (A) HUVECs infected with Ad-SIRT1 or control Ad-GFP were pretreated with NFAT inhibitor (30 µg/ml) or NF-κB inhibitor JSH-23 (30 µM) for 1 h and then incubated with PMA (10 ng/ml) and Io (0.25 µM) or vehicle DMSO for another 3 h. Total cell extracts were analyzed for COX-2 by western blotting. The images are representative of three independent experiments. (B) HUVECs were infected with Ad-SIRT1 RNAi or control Ad-U6, and then incubated with PMA (10 ng/ml) and Io (0.25 µM), or vehicle DMSO for 3 h. Total cell extracts were analyzed for COX-2 by western blotting. The images are representative of three independent experiments. (C, E) HUVECs were pretreated with resveratrol (RSV, 50 µM) or vehicle DMSO for 1 h and then incubated with PMA (10 ng/ml) and Io (0.25 µM) or vehicle DMSO for another 3 h. Total RNA and protein extracts were analyzed for COX-2 by real-time PCR (C) and western blotting (E), respectively. The images are representative of three independent experiments. (D, E) HUVECs were treated with SIRT1 inhibitor Sirtinol (30 µM) or vehicle DMSO for 1 h. Total RNA and protein extracts were analyzed for COX-2 by real-time PCR (D) and western blotting (E). The images are representative of three independent experiments.

NFAT plays an essential role in the regulation of the expression of the COX-2 gene [26]. To detect whether NFAT is involved in the inhibition of COX-2 expression by SIRT1 in PMA/Io-treated HUVECs, we infected HUVECs with Ad-SIRT1 followed by pretreatment with NFAT inhibitor and then treatment with PMA/Io for 3 h. We found that over-expression of SIRT1 suppressed COX-2 expression in PMA/Io-treated HUVECs, and that inhibition of COX-2 expression by SIRT1 in PMA/Io-treated HUVECs was largely abrogated by inhibiting NFAT activation (Figure 5A). Interestingly, NF-κB inhibitor JSH-23 also largely abrogated inhibition of COX-2 expression by SIRT1 in PMA/Io-treated HUVECs (Figure 5A). To detect the effect of SIRT1 knockdown on COX-2 expression in endothelial cells, we infected HUVECs with Ad-SIRT1 RNAi followed by treatment with PMA/Io. Knockdown of SIRT1 caused upregulation of COX-2 expression in the absence and presence of PMA/Io-treated HUVECs (Figure 5B).

Furthermore, we pretreated HUVECs with SIRT1 activator Resveratrol (RSV) followed by treatment with PMA/Io for 3 h. RSV significantly reduced COX-2 mRNA (Figure 5C) and protein expression (Figure 5E) in PMA/Io-stimulated HUVECs. We also examined the effect of SIRT1 inhibitor Sirtinol on the expression of COX-2 in HUVECs. Sirtinol significantly induced COX-2 mRNA (Figure 5D) and protein expression (Figure 5E) in HUVECs.

### SIRT1 suppresses NFAT-induced COX-2 promoter activity and reduces NFAT binding to the promoter of COX-2

**Figure 6:**
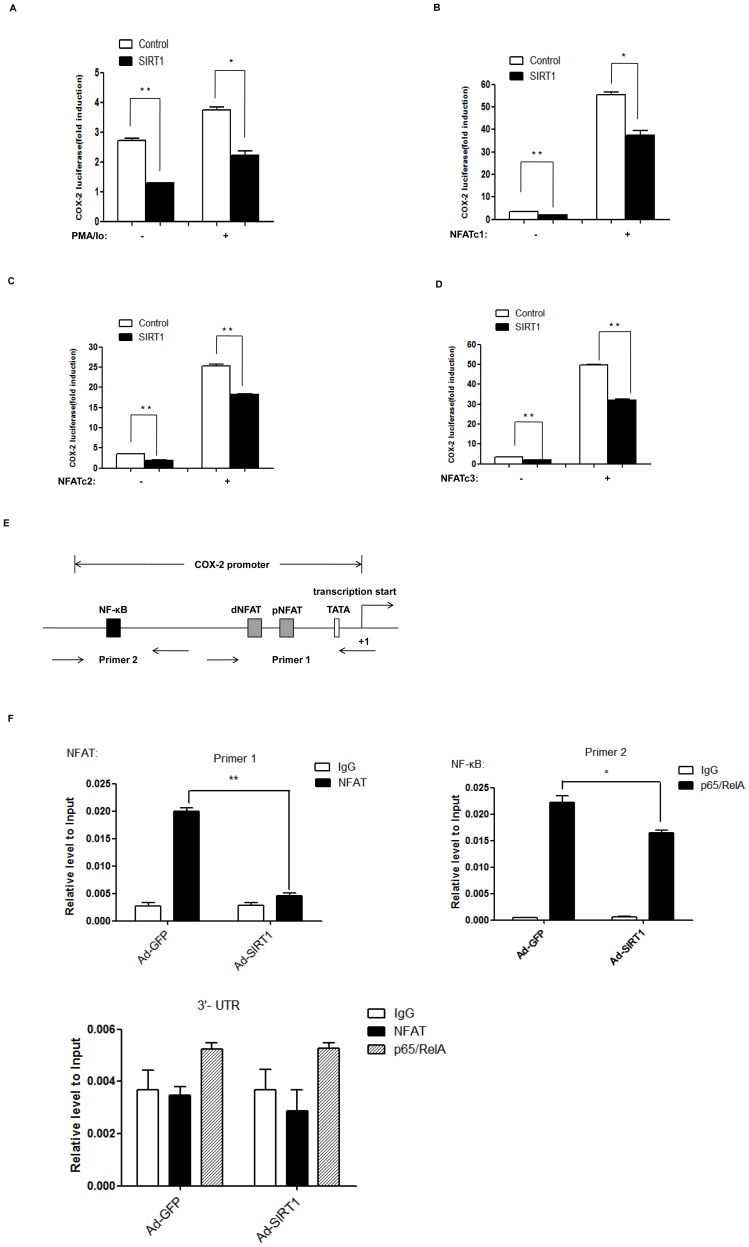
SIRT1 suppresses NFAT-induced COX-2 promoter activity and reduces NFAT binding to the promoter of COX-2. (A) HEK293 cells were transfected with 0.1 µg COX-2 luciferase reporter (COX-2 -luc), 30 ng pRL-TK, and 0.3 µg SIRT1 expression vector or control (pcDNA3.1) vectors for 24 h and were treated with PMA (10 ng/ml) and Io (0.25 µM), or vehicle DMSO for another 3 h. Luciferase activities are presented as the means ± standard deviation (S.D.) of triplicate samples and are representative of three independent experiments. **, p≤0.01 versus control. *, p≤0.05 versus control. (B–D) HEK293 cells were transfected with 0.1 µg COX-2 luciferase reporter (COX-2 -luc), 30 ng pRL-TK, 0.3 µg SIRT1 expression vector or control (pcDNA3.1) vector, and 0.3 µg NFATc1 (B), or NFATc2 (C), or NFATc3 (D) for 24 h. Luciferase activities are presented as the means ± standard deviation (S.D.) of triplicate samples and are representative of three independent experiments.**, p≤0.01 versus control. *, p≤0.05 versus control. (E) Diagrammatic drawing shows the binding sites of NFAT and NF-κB on human COX-2 promoter. There are two NFAT cis-acting elements named NFAT distal (dNFAT, −102 to −96 bp) and NFAT proximal (pNFAT, −73 to −67 bp) elements locating upstream of the transcription starting site of COX-2. There is a NF-κB cis-acting element (−447 to −439 bp) locating upstream of the transcription starting site of COX-2. And the designed primers covered these sites are shown. (F) HUVECs were infected with Ad-SIRT1 or control Ad-GFP for 24 h, and then were treated with PMA (10 ng/ml) and Io (0.25 µM) for 3 h. ChIP assays were performed with chromatin prepared from HUVECs. Chromatin was immunoprecipitated with normal rabbit IgG, antibody against NFATc3 or NF-κB p65, and precipitated genomic DNA was analyzed by real-time PCR using primers for the NFAT or NF-κB p65 binding site on COX-2 promoter or 3′-UTR region of COX-2 gene. Data was shown as the mean ± standard deviation (S.D.) for three independent experiments. **, p≤0.01 versus indicated group.*, p≤0.05 versus indicated group.

To examine the effect of SIRT1 on COX-2 promoter activity, HEK293 cells were co-transfected with the COX-2 promoter reporter (COX-2 luc), pRL-TK (internal control) and SIRT1 expression vector followed by treatment with PMA/Io. The luciferase reporter assay showed that PMA/Io induced COX-2 promoter activity, while SIRT1 suppressed PMA/Io-induced COX-2 promoter activity (Figure 6A). To further determine whether the suppressive effect of SIRT1 on NFAT transcriptional activity results in down-regulation of COX-2 gene transcription, HEK293 cells were transfected with the COX-2 promoter reporter (COX-2 luc), pRL-TK (internal control), NFAT and SIRT1 expression vectors. NFAT induced COX-2 promoter activity, while SIRT1 suppressed NFAT induced COX-2 promoter activity (Figure 6B, 6C and 6D).

Iniguez et al have identified two NFAT bindingsites in the 5′-GGGAAAA-3′(−102 to −96 bp) and 5′-CGGAAAG-3′ (−73 to −67 bp) from the transcription start site required for induction of human COX-2 promoter activity [26]. In addition, a NF-κB binding site also exists in human COX-2 promoter region: 5′-GGGATTCCC-3′ (−447 to −439 bp) [27], [28]. Schematic diagram of two NFAT binding sites and one NF-κB binding site on human COX-2 promoter is shown in Figure 6E. To examine whether SIRT1 influences NFAT or NF-κB p65 binding to the COX-2 promoter in PMA/Io-treated HUVECs, we performed ChIP assays in HUVECs infected with Ad-SIRT1 followed by treatment with PMA/Io. We found that SIRT1 inhibited both NFAT and NF-κB p65 binding to the COX-2 promoter in PMA/Io-treated HUVECs (Figure 6F).

## Discussion

HDACs act as important cofactors that are recruited by transcription factors to regulate chromatin remodeling, transcription factor activity, and gene transcription [29], [30]. NFAT plays a pivotal role in the transcription of genes critical for inflammatory responses [31]. The results of the present study revealed a key role of the class III HDAC SIRT1 in the regulation of transcriptional activity of NFAT. We found that SIRT1 suppressed NFAT transcriptional activity, and interacted with NFAT. Moreover, SIRT1 primarily deacetylated NFATc3. Inhibition of COX-2 expression by SIRT1 in PMA/Io-treated HUVECs was largely abrogated by inhibiting NFAT activation. Furthermore, SIRT1 inhibited NFAT induced COX-2 promoter activity, and reduced NFAT binding to the COX-2 promoter in PMA/Io-treated HUVECs. The suppressive effect of SIRT1 on COX-2 expression was mediated at least in part by regulating the transcription factor NFAT. These findings indicate that the negative regulating mechanisms of NFAT by SIRT1 may contribute to its anti-inflammatory effect in atherosclerosis.

SIRT1 prevented endothelial superoxide production, inhibited NF-κB signaling, and diminished expression of adhesion molecules [19]. SIRT2 down-regulates expression of genes involved in immune and inflammatory response such as COX-2 through deacetylation of p65 Lys310 in an NF-κB-dependent manner [32]. Here, we found that SIRT1 but not SIRT2 (Figure S2) suppressed NFAT transcriptional activity. The finding implies that the inhibitory effect of SIRT1 on NFAT transcriptional activity is sirtuin family member-specific.

The promoter region of the COX-2 gene contains binding sites for transcription factors including NF-κB [27] and NFAT [26]. We found that SIRT1 inhibited PMA/Io-induced NF-κB [33] and NFAT transcriptional activity. Moreover, inhibition of COX-2 expression by SIRT1 in PMA/Io-treated HUVECs was largely abrogated by inhibiting NFAT or NF-κB activation. The results indicated that both NF-κB and NFAT were involved in the inhibition of COX-2 expression by SIRT1 in PMA/Io-treated HUVECs. In addition, SIRT1 suppressed NFAT-induced COX-2 promoter activity, and inhibited NFAT binding to the COX-2 promoter in PMA/Io-treated HUVECs. Similarly, SIRT1 also inhibited NF-κB p65 DNA binding ability on the COX-2 promoter in PMA/Io-treated HUVECs. The data suggest that the suppressive effect of SIRT1 on NFAT and NF-κB p65 DNA binding ability results in reduced COX-2 promoter activity in PMA/Io-treated HUVECs.

SIRT1 suppresses AP-1 transcriptional activity and COX-2 expression in macrophages [18]. AP-1 is the most important partner of NFAT in transcription [25]. Composite NFAT:AP-1 sites have been described in numerous genes involved in the productive immune response, such as COX-2 [25]. Consistent with the previous report, NFATc3 is activated and nuclear translocated in PMA/Io-stimulated HUVECs, and NFAT inhibitor CsA blocked COX-2 expression induced by PMA/Io (Figure S4). Moreover, SIRT1 suppressed PMA/Io-induced COX-2 expression in HUVECs. Here, we found that SIRT1 did not interfere the interaction between NFAT and AP-1 (c-Jun and c-Fos). It suggests that the SIRT1-NFAT-COX-2 signal pathway is not dependent on AP-1.

SIRT1 mediates the anti-oxidative and anti-inflammatory vasoprotective effects of caloric restriction in aging [34]. SIRT1 is highly expressed in the vasculature and acts as a key regulator of vascular endothelial homeostasis controlling angiogenesis, vascular tone and endothelial dysfunction [11], [12]. NFAT proteins, which are expressed in most immune-system cells, play a pivotal role in the transcription of cytokine genes and other genes critical for the immune response [31]. NFATc3 isoform is specifically implicated in vasculature development [35], maintenance of a contractile phenotype [36] and regulation of vascular smooth muscle cell (VSMC) contractility [37]. The biological functions of SIRT1 are thought to be mediated by its deacetylase activity [38]. Acetylation level could contribute to activity of transcriptional factor, such as p53, by affecting its DNA binding [39]. The SIRT1 deacetylase binds to and deacetylates NF-κB p65 at lysine 310, thereby inhibiting NF-κB-mediated transcription [40]. Our data showed that SIRT1 notably reduced the acetylation level and transcription activity of NFATc3. SIRT1 reduced NFAT binding to the COX-2 promoter in PMA/Io-treated HUVECs. Moreover, inhibition of COX-2 expression by SIRT1 in PMA/Io-treated HUVECs was largely abrogated by inhibiting NFAT activation. However, it is still unclear whether deacetylation of NFATc3 by SIRT1 mediates the suppressive effects of SIRT1 on COX-2 expression in PMA/Io-treated HUVECs, which needs further investigation. Taken together, these data suggest that SIRT1 regulates endothelial angiogenic, contractile and inflammatory functions, at least in part, by modulating the transcriptional activity of NFATc3.

In conclusion, the findings in the present study expand the role of SIRT1 as a critical regulator of inflammation by functioning as a transcriptional suppressor of NFAT. Thus, these findings indicate inhibition of NFAT by SIRT1 might contribute to alleviating inflammation for vascular-regulated disease such as atherosclerosis.

## Supporting Information

Figure S1 **NFAT isoforms expression in ECV304.** Total RNA was isolated from Jurkat cells and human umbilical vein endothelial cell line ECV304, and mRNA levels for NFATc1, NFATc2, NFATc3 and NFATc4 were analyzed by reverse transcription PCR. The images are representative of three independent experiments.
(TIF)

Figure S2 **SIRT2 does not affect NFAT transcriptional activity.** HEK293 cells were transfected with 0.1 µg NFAT luciferase reporter (NFAT-luc), 30 ng pRL-TK, 0.3 µg NFATc1 (A) or NFATc2 (B) or NFATc3 (C), and 0.3 µg SIRT2 or control (pcDNA3.1) for 24 h. Luciferase activities are presented as the means ± standard deviation (S.D.) of triplicate samples and are representative of three independent experiments.
(TIF)

Figure S3 **SIRT1 had no obvious influence on acetylation level of NFATc1 and NFATc2.** HEK293 cells were transfected with: (A) HA-tagged NFATc1 and either SIRT1 or control (pcDNA3.1) vectors for 24 h; (B) HA-tagged NFATc2 and either SIRT1 or control (pcDNA3.1) vectors for 24 h. HA-tagged NFATc1 or NFATc2 was immunoprecipitated and the acetylation of NFATc1 or NFATc2 was assessed by western blotting using anti-acetylated lysine antibody. Total levels of NFATc1 or NFATc2 were assessed with anti-HA antibody.
(TIF)

Figure S4 **NFAT mediates PMA/Io induced COX-2 expression in HUVECs.** (A) HUVECs were treated with PMA (10 ng/ml) alone, Io (0.25 µM) alone, PMA (10 ng/ml) and Io (0.25 µM) for 3 h. Total RNA was isolated and levels for COX-2 were analyzed by real-time PCR. The images are representatives of three independent experiments. (B) HUVECs were incubated with CsA (1 µM) for 1 h prior treatement with PMA (10 ng/ml) alone, Io (0.25 µM) alone, PMA (10 ng/ml) and Io (0.25 µM) for 3 h. Amounts of COX-2 protein in the total cell extracts of HUVECs were assessed by western blotting. The images are representatives of three independent experiments. (C) HUVECs were pretreated with CsA (1 µM) for 1 h, then treated with PMA (10 ng/ml) and Io (0.25 µM) for another 3 h.Total RNA was isolated and levels for COX-2 were analyzed by real-time PCR. The images are representatives of three independent experiments.
(TIF)

Table S1 **PCR primer used for generation of HA-NFAT.**
(TIF)

Table S2 **PCR primer used for generation of Myc-NFAT.**
(TIF)

Table S3 **PCR primer used for generation of truncated HA-NFATc3.**
(TIF)

Table S4 **Real-time PCR primer used for mRNA expression of COX-2 and β-Actin.**
(TIF)

Table S5 **Real-time primer used for ChIP on COX-2 promoter or 3′-UTR.**
(TIF)
