# Supplementary figures and images for: The Involvement of NFAT Transcriptional Activity Suppression in SIRT1-Mediated Inhibition of COX-2 Expression Induced by PMA/Ionomycin
Source: PLoS One. 2014 May 23;9(5):e97999. doi: 10.1371/journal.pone.0097999 (PMC4032329; doi:10.1371/journal.pone.0097999)

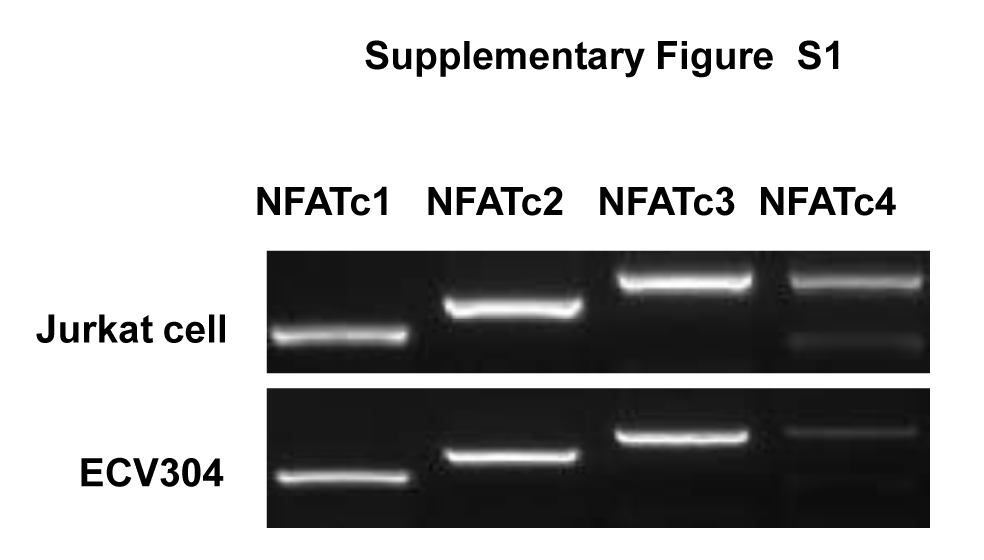

Supplement: Figure S1 — NFAT isoforms expression in ECV304. Total RNA was isolated from Jurkat cells and human umbilical vein endothelial cell line ECV304, and mRNA levels for NFATc1, NFATc2, NFATc3 and NFATc4 were analyzed by reverse transcription PCR. The images are representative of three independent experiments. (TIF) [file pone.0097999.s001.tif]

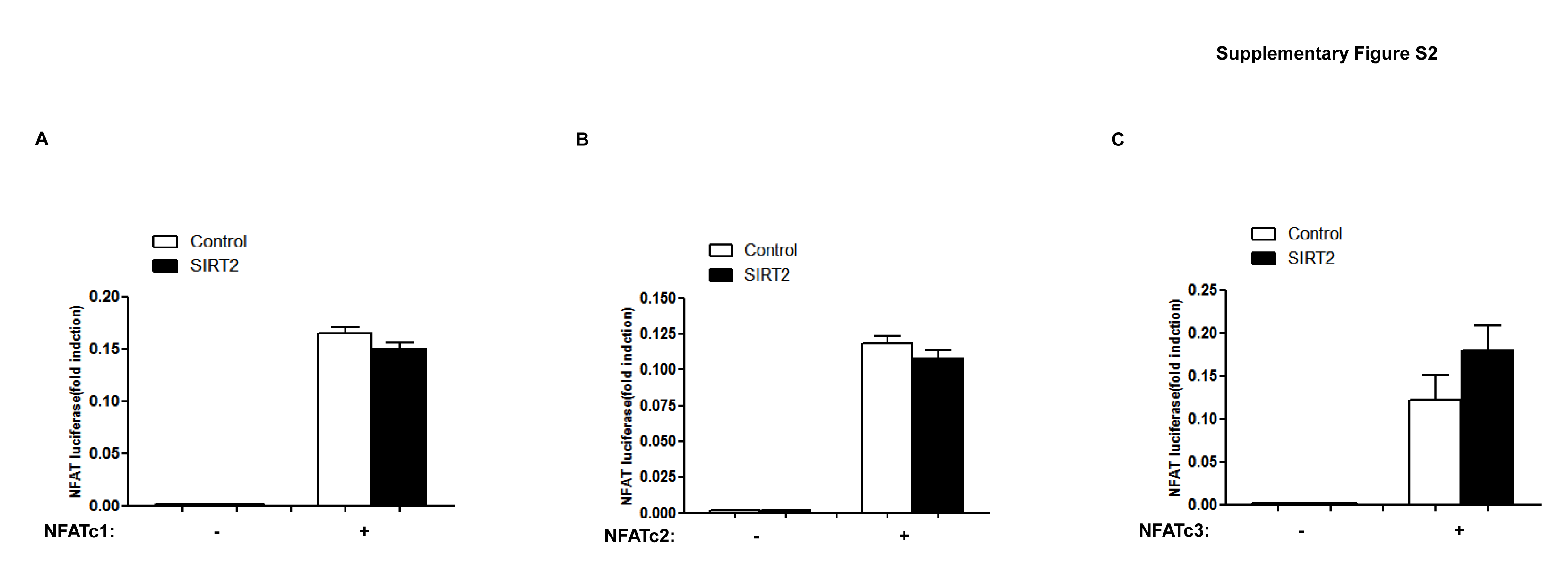

Supplement: Figure S2 — SIRT2 does not affect NFAT transcriptional activity. HEK293 cells were transfected with 0.1 µg NFAT luciferase reporter (NFAT-luc), 30 ng pRL-TK, 0.3 µg NFATc1 (A) or NFATc2 (B) or NFATc3 (C), and 0.3 µg SIRT2 or control (pcDNA3.1) for 24 h. Luciferase activities are presented as the means ± standard deviation (S.D.) of triplicate samples and are representative of three independent experiments. (TIF) [file pone.0097999.s002.tif]

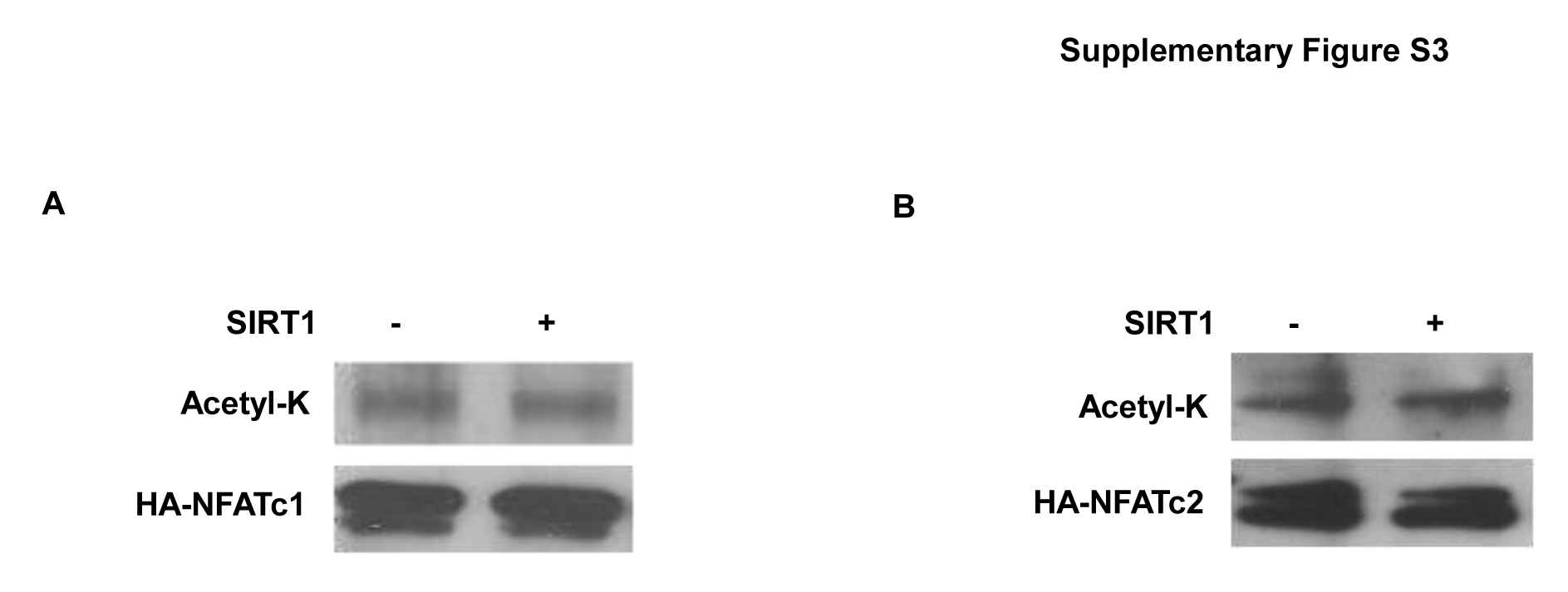

Supplement: Figure S3 — SIRT1 had no obvious influence on acetylation level of NFATc1 and NFATc2. HEK293 cells were transfected with: (A) HA-tagged NFATc1 and either SIRT1 or control (pcDNA3.1) vectors for 24 h; (B) HA-tagged NFATc2 and either SIRT1 or control (pcDNA3.1) vectors for 24 h. HA-tagged NFATc1 or NFATc2 was immunoprecipitated and the acetylation of NFATc1 or NFATc2 was assessed by western blotting using anti-acetylated lysine antibody. Total levels of NFATc1 or NFATc2 were assessed with anti-HA antibody. (TIF) [file pone.0097999.s003.tif]

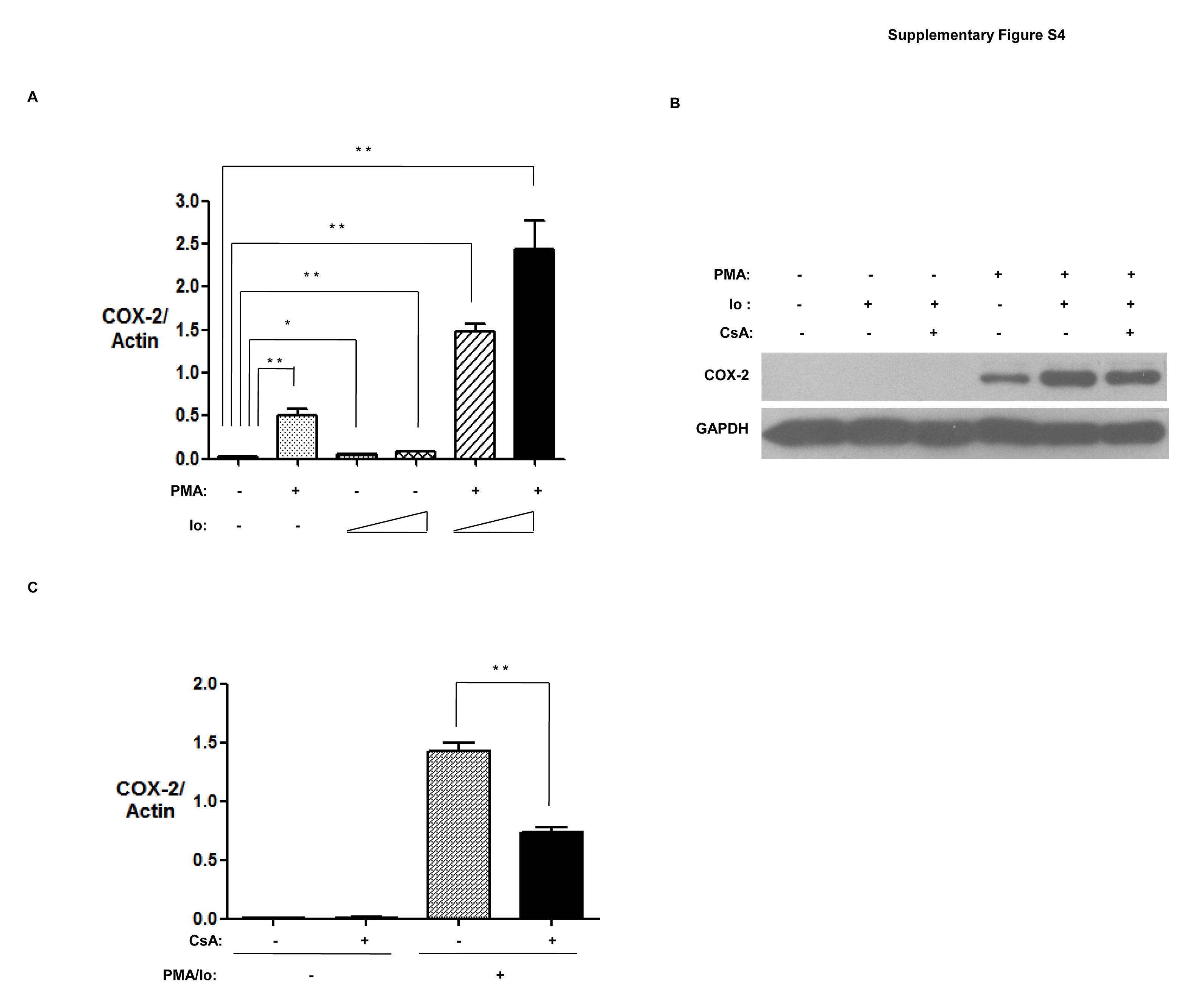

Supplement: Figure S4 — NFAT mediates PMA/Io induced COX-2 expression in HUVECs. (A) HUVECs were treated with PMA (10 ng/ml) alone, Io (0.25 µM) alone, PMA (10 ng/ml) and Io (0.25 µM) for 3 h. Total RNA was isolated and levels for COX-2 were analyzed by real-time PCR. The images are representatives of three independent experiments. (B) HUVECs were incubated with CsA (1 µM) for 1 h prior treatement with PMA (10 ng/ml) alone, Io (0.25 µM) alone, PMA (10 ng/ml) and Io (0.25 µM) for 3 h. Amounts of COX-2 protein in the total cell extracts of HUVECs were assessed by western blotting. The images are representatives of three independent experiments. (C) HUVECs were pretreated with CsA (1 µM) for 1 h, then treated with PMA (10 ng/ml) and Io (0.25 µM) for another 3 h.Total RNA was isolated and levels for COX-2 were analyzed by real-time PCR. The images are representatives of three independent experiments. (TIF) [file pone.0097999.s004.tif]

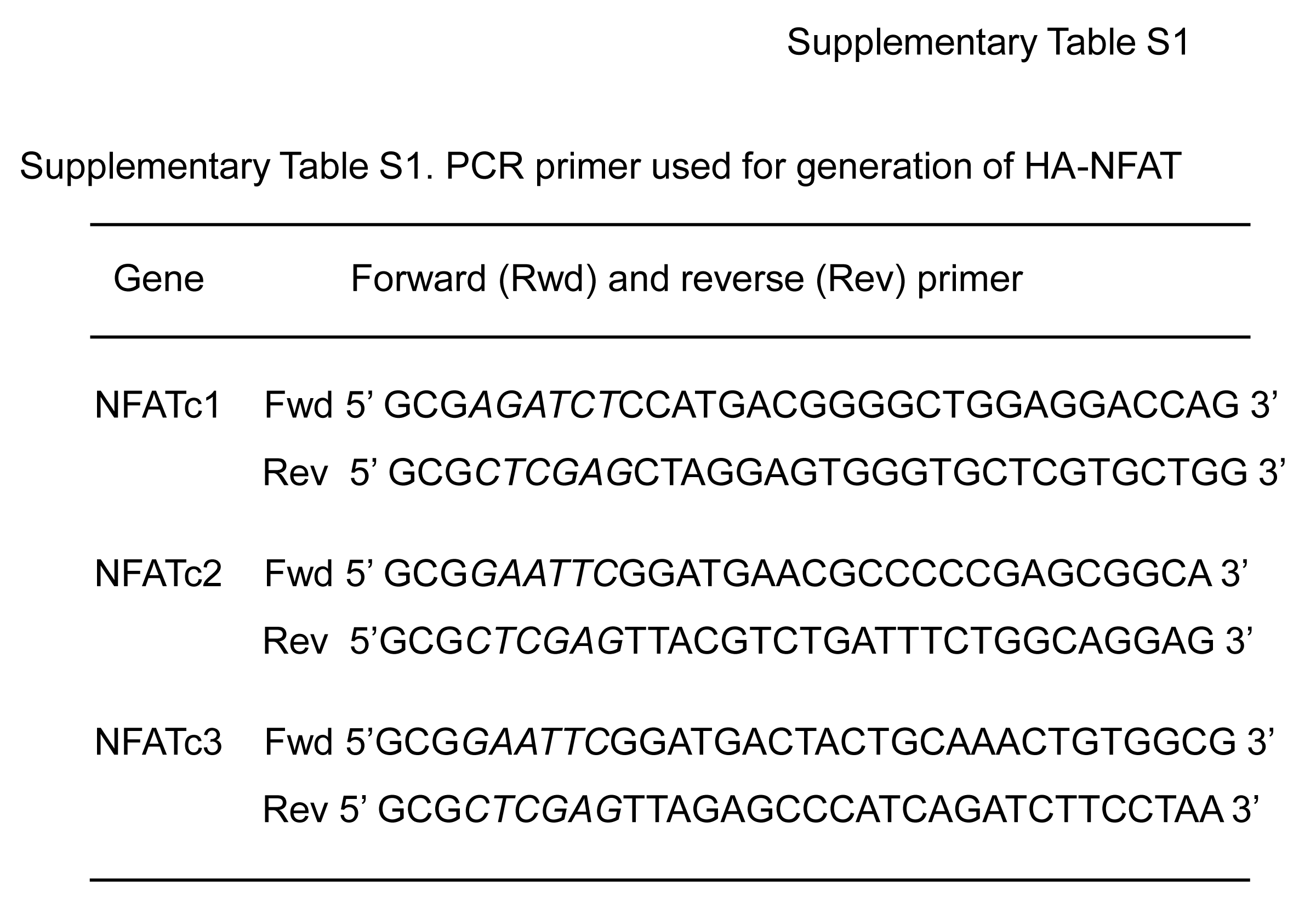

Supplement: Table S1 — PCR primer used for generation of HA-NFAT. (TIF) [file pone.0097999.s005.tif]

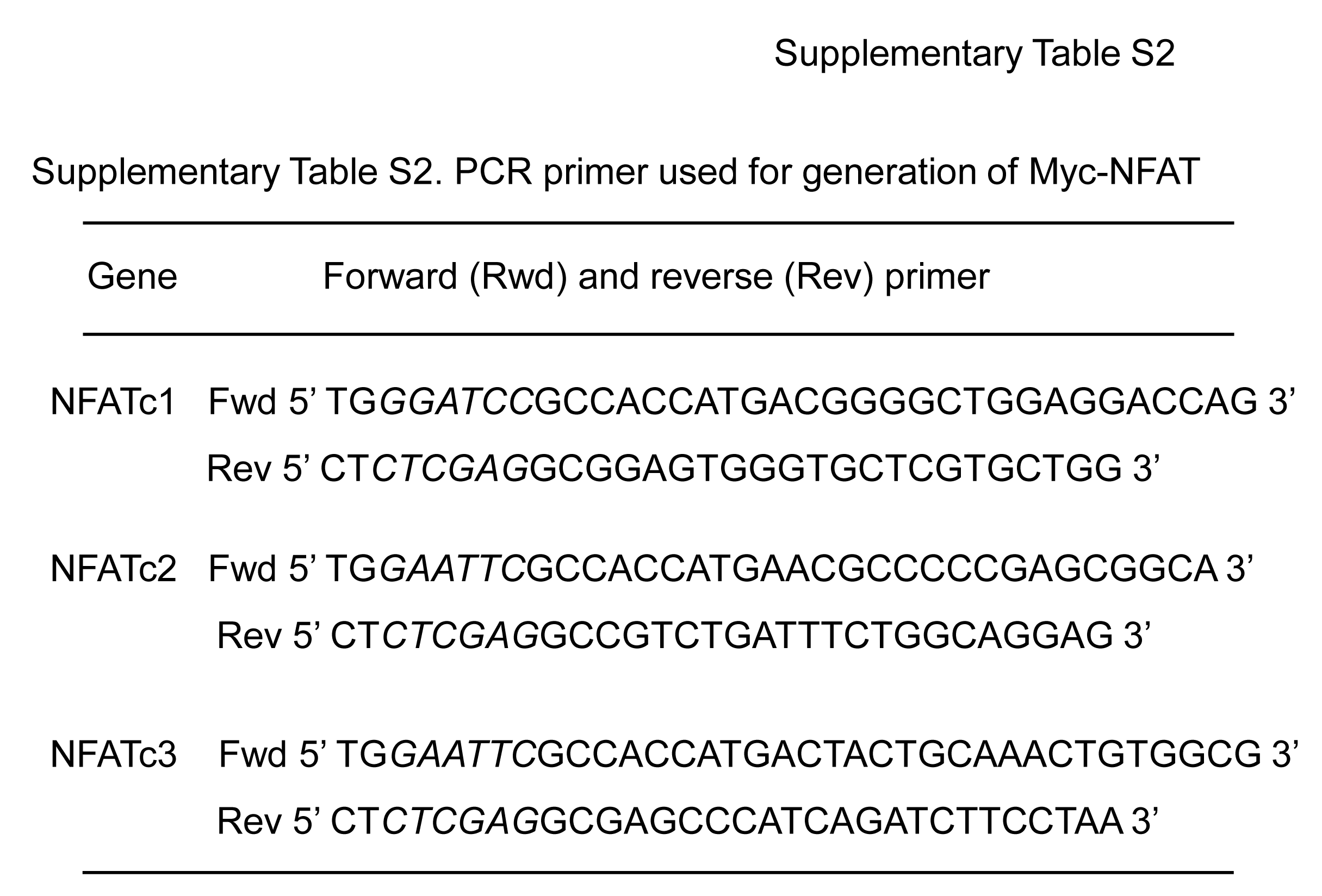

Supplement: Table S2 — PCR primer used for generation of Myc-NFAT. (TIF) [file pone.0097999.s006.tif]

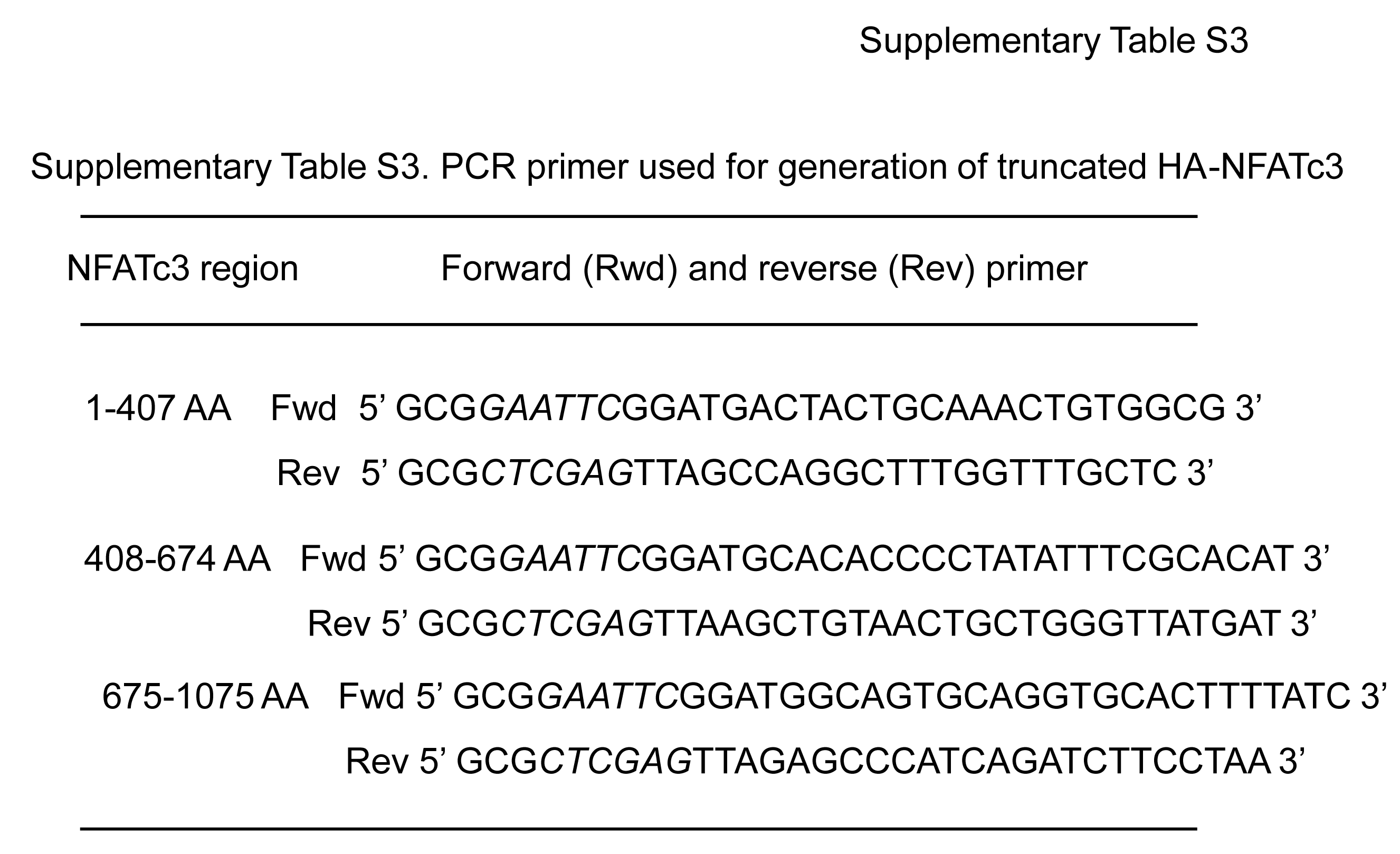

Supplement: Table S3 — PCR primer used for generation of truncated HA-NFATc3. (TIF) [file pone.0097999.s007.tif]

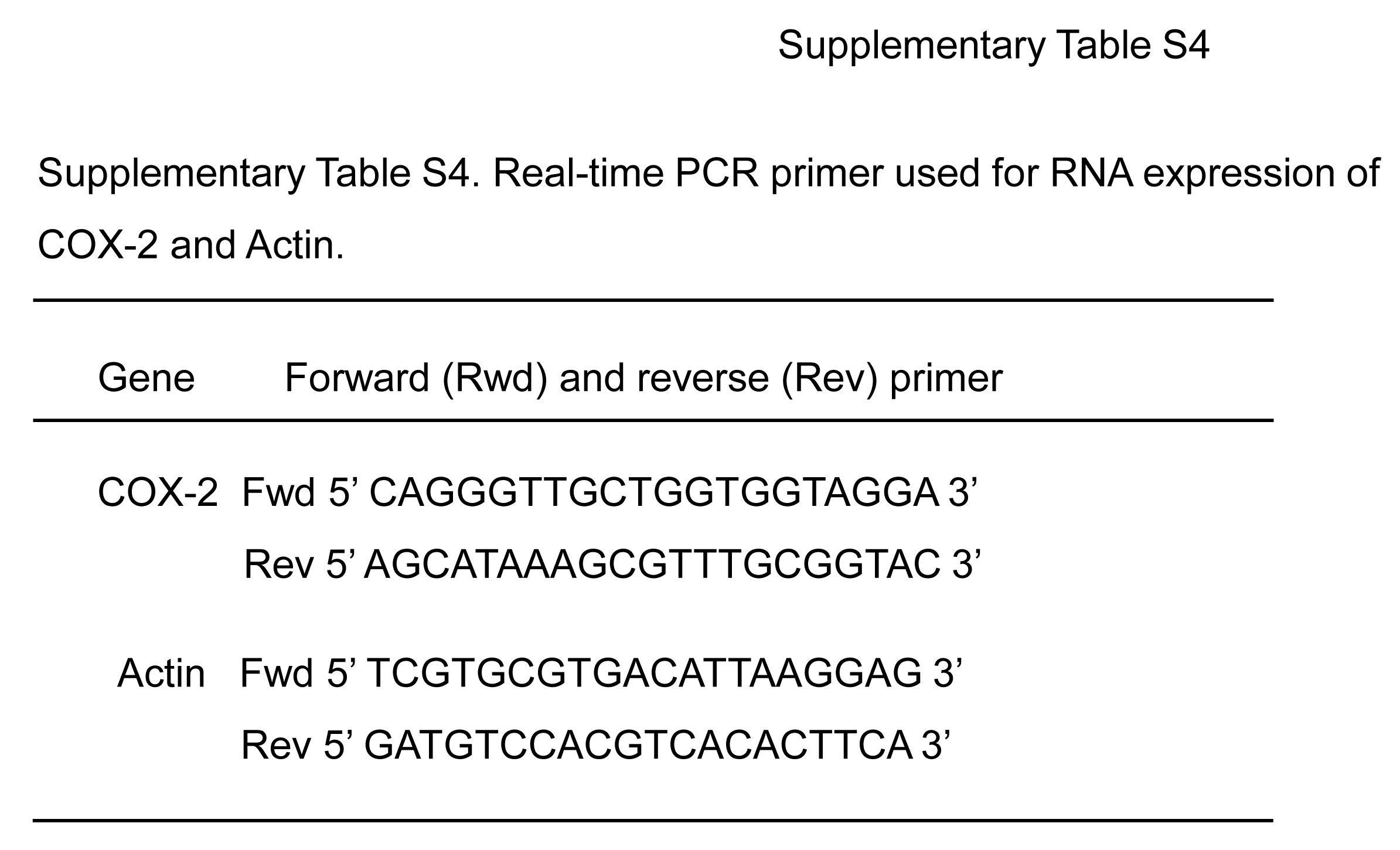

Supplement: Table S4 — Real-time PCR primer used for mRNA expression of COX-2 and β-Actin. (TIF) [file pone.0097999.s008.tif]

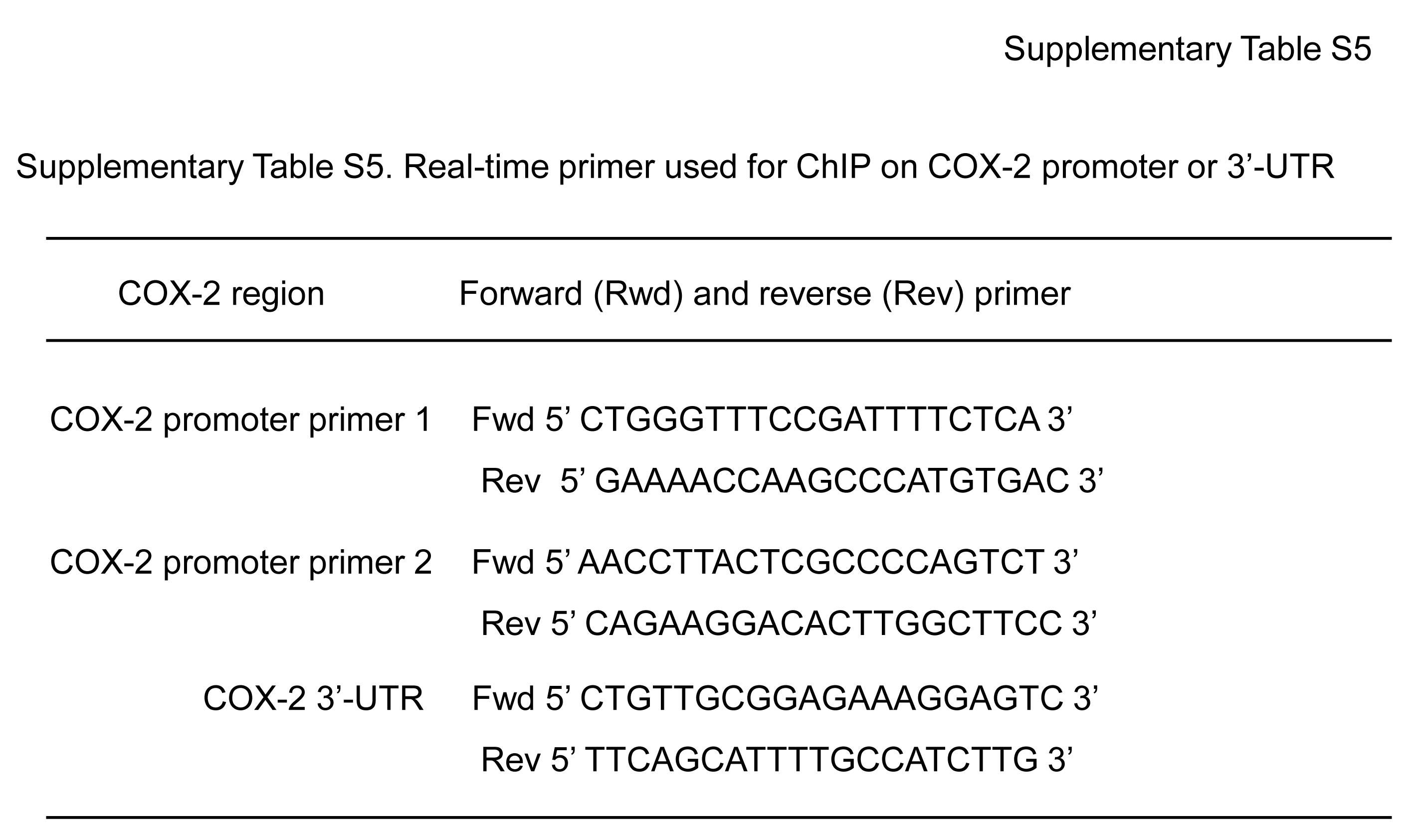

Supplement: Table S5 — Real-time primer used for ChIP on COX-2 promoter or 3′-UTR. (TIF) [file pone.0097999.s009.tif]
